# Biventricular circulatory support using single-device and dual-device configurations: Initial pump characterization in simulated heart failure model

**DOI:** 10.3389/fcvm.2023.1045656

**Published:** 2023-02-22

**Authors:** Jamshid H. Karimov, Chihiro Miyagi, Christine R. Flick, Anthony R. Polakowski, Barry D. Kuban, Taiyo Kuroda, Dennis W. Horvath, Kiyotaka Fukamachi, Randall C. Starling

**Affiliations:** ^1^Department of Biomedical Engineering, Lerner Research Institute, Cleveland Clinic, Cleveland, OH, United States; ^2^Cleveland Clinic Lerner College of Medicine of Case Western Reserve University, Cleveland Clinic, Cleveland, OH, United States; ^3^R1 Engineering LLC, Euclid, OH, United States; ^4^Department of Cardiovascular Medicine, Miller Family Heart and Vascular Institute, Cleveland Clinic, Cleveland, OH, United States; ^5^Kaufman Center for Heart Failure Treatment and Recovery, Cleveland Clinic, Cleveland, OH, United States

**Keywords:** mechanical circulatory support, biventricular failure, blood pumps, ventricular assist device, total artificial heart, universal device

## Abstract

**Objective:**

Severe biventricular heart failure (BHF) can be remedied using a biventricular assist device (BVAD). Two devices are currently in development: a universal ventricular assist device (UVAD), which will be able to assist either the left, right, or both ventricles, and a continuous-flow total artificial heart (CFTAH), which replaces the entire heart. In this study, the *in vitro* hemodynamic performances of two UVADs are compared to a CFTAH acting as a BVAD.

**Methods:**

For this experiment, a biventricular mock circulatory loop utilizes two pneumatic pumps (Abiomed AB5000™, Danvers, MA, USA), in conjunction with a dual-output driver, to create heart failure (HF) conditions (left, LHF; right, RHF; biventricular, BHF). Systolic BHF for four different situations were replicated. In each situation, CFTAH and UVAD devices were installed and operated at two distinct speeds, and cannulations for ventricular and atrial connections were evaluated.

**Results:**

Both CFTAH and UVAD setups achieved our recommended hemodynamic criteria. The dual-UVAD arrangement yielded a better atrial balance to alleviate LHF and RHF. For moderate and severe BHF scenarios, CFTAH and dual UVADs both created excellent atrial pressure balance. Conversely, when CFTAH was atrial cannulated for LHF and RHF, the needed atrial pressure balance was not met.

**Conclusion:**

Comprehensive *in vitro* testing of two different BVAD setups exhibited self-regulation and exceptional pump performance for both (single- and dual-device) BHF support scenarios. For treating moderate and severe BHF, UVAD and CFTAH both functioned well with respect to atrial pressure regulation and cardiac output. Though, the dual-UVAD setup yielded a better atrial pressure balance in all BHF testing scenarios.

## 1. Introduction

Worldwide, approximately 26 million people are affected by Heart failure (HF), which significantly affects cardiovascular mortality (1). Heart transplants notably improve the quality of life and the survival rate for patients (2, 3), however, a scarcity of donor hearts continuously limits this treatment option (4). In the USA, an estimated 50,000–100,000 patients require heart transplants or treatment with mechanical circulatory support (MCS), but annually, only ∼3,500 patients receive the ultimate life changer, a heart transplant. as an alternative, patients may instead receive left ventricular assist devices (LVADs), which is the traditional treatment to correct hemodynamic levels for both transplant-eligible, and non-eligible, patients in organ decompensation (3).

In recent years, LVAD technology has decisively switched to continuous flow (CF) rotary pump designs, and shifted away from the old-style, volume-displacement pulsatile-flow devices. Due to better mechanical reliability, greater durability, and being more compact in size, greater than 95% of device implants utilized today are CF-type designs. However, despite recent progress with LVADs, the demand for durable right ventricular support remains unmet (5). The cumulative experience with commercially available LVADs for right ventricular recovery has been variable (6), and there is still disagreement across data sources with regard to device selection, management and recovery assessment post-right ventricular assist device (RVAD) implant.

Biventricular heart failure (BHF) patients are extremely ill, and may suffer from end-stage cardiac disease affecting both ventricles (even though initial onset may have been a typical left-side heart disease) (7). Unfortunately, patients with biventricular assist devices (BVADs) experience more adverse events and poor 1 year survival rates (8, 9). BHF necessitates timely treatment to support worsening hemodynamic levels, and to enable the patient to recover (9). With the observed evolution of LVADs, a shift in patient phenotypes (10, 11), and a steady rise in HF occurring in older patients experiencing more comorbidities, it can be concluded that durable biventricular MCS is in high demand now, and will be for the foreseeable future (6). Currently, there are no durable MCS technologies approved for biventricular support, but selected device-based options are being explored for their capability to provide support for BHF (12–14).

In this study, a Cleveland Clinic CF total artificial heart (CFTAH) and two universal ventricular assist devices (UVADs) are evaluated for circulatory support, both systemic and pulmonary, and we provide a first extensive review of our *in vitro* assessment data while simulating single- and dual-device BVAD support for BHF. Pump performance, physiologic and hemodynamic parameters, as well as the advantages and limitations of each BVAD configuration option, are presented.

## 2. Materials and methods

### 2.1. Biventricular mock circulatory loop

**FIGURE 1:**
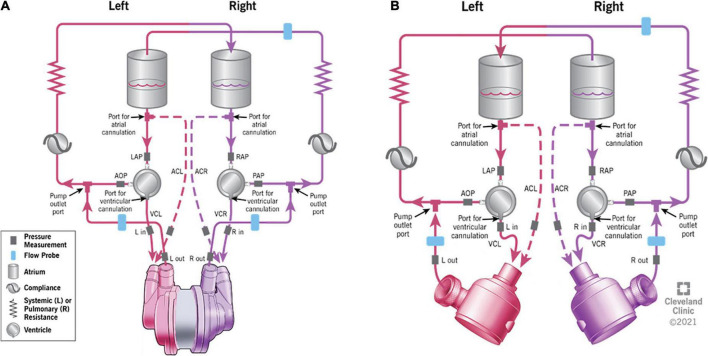
The schematic representation of the testing mock loop specifically designed for biventricular assist device (BVAD) device testing. **(A)** – continuous-flow total artificial heart (CFTAH); **(B)** – dual universal ventricular assist device (UVAD). Ventricular cannulation (VC) and atrial cannulation (AC) options are shown. Dashed lines indicate the optional atrial cannulation on the mock circulatory loop.

**FIGURE 2:**
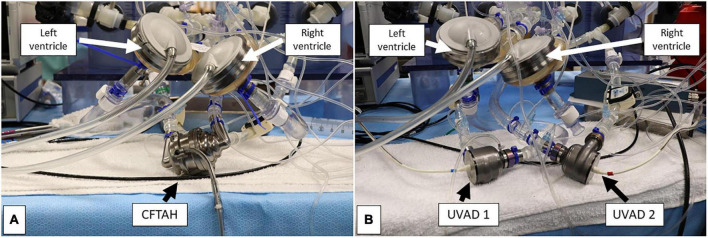
Bench view of the single-device **(A)** and dual-device **(B)** pump configurations during assessment. Both biventricular assist device (BVAD) configurations were connected to the respective device inflow and outflow ports.

Both devices configurations were individually and consecutively tested on the same testing loop (Figure 1). The conditions for the study were simulated by using a test loop specifically configured for biventricular testing (15), which has been used previously for characterization of other CF devices and disease states. The test loop has two pneumatic pumps (Abiomed AB5000, Danvers, MA, USA) powered from a dual-output driver (Figure 2). The working fluid in the test loop was a blood analog glycerin/water mixture with a specific gravity of 1.060. Manual valves simulated vascular resistances, closed pneumatic reservoirs created arterial compliances, and pump inlets were filled by open reservoirs. Inflows for both BVADs were connected to the loop in both ventricular and atrial positions. Adjustable parameters for the loop included: fluid volumes, drive pressures, beat rates for pumps, loop compliance, vascular resistance (systemic and pulmonary), and shunt flows between all four chambers. For this study, we varied the drive pressures to simulate four different conditions: moderate left heart failure (LHF), moderate right heart failure (RHF), moderate biventricular failure (BHF), and severe BHF (Table 1).

**TABLE 1:** Filling systolic and vacuum diastolic pneumatic drive pressures for mock ventricles.

Ventricles	Left		Right	
Pump parameters	Driving pressure	Vacuum	Driving pressure	Vacuum
	mm Hg		mm Hg	
LHF	130	-40	130	-20
RHF	200	-40	105	-20
BHF	130	-40	105	-20
Severe BHF	70	-40	75	-20

LHF, left heart failure; RHF, right heart failure; BHF, biventricular heart failure.

### 2.2. Device descriptions

Both the CFTAH and the UVAD are CF pumps with a single motor and single rotor that were designed to enable their intrapericardial implantation. The devices are intended as durable MCS, and each is managed by its dedicated controller unit.

#### 2.2.1. CFTAH

The Cleveland Clinic CFTAH has two inflow and two outflow ports, and provides both systemic and pulmonary circulatory support in a single assembly with one moving part running at the same speed: a double-ended rotor with two impellers, uniquely designed for right (pulmonary) and left (systemic) circulatory support (16). The CFTAH, when used as a total artificial heart, passively self-regulates relative left and right output to achieve systemic and pulmonary fluid balance. To enable the self-regulation feature, CFTAH has passive magnetic forces created between the motor’s steel laminations and magnets inside the rotor, which stabilizes the rotor axially, and blood creates a hydrodynamic journal bearing that radially suspends the rotor (17). CFTAH has a unique aperture, which acts as a valve that is regulated by differential pressure. This feature automatically adjusts left and right inlet pressures. This aperture creates added flow resistance, which regulates left and right pump hydraulic pressures required for higher-pressure systemic, and lower-pressure pulmonary, circulations, respectively (18).

Both pump impellers have extra clearance at each end to allow passive rotor movement axially. The axial movement of the rotor opens and closes the aperture located near the right pump’s outlet, regulating pulmonary flow, which regulates the balance of pressures across the right and left sides. For this study, the CFTAH was run at two different speeds (3,000 and 3,500 rpm).

#### 2.2.2. UVAD

The UVAD is a CF pump that incorporates an aperture feature (similar to the CFTAH’s right pump), which actuates passively in response to the pressures at the inlet and outlet of the pump. A weak axial magnetic force pushes the rotor toward a closed aperture, restricting backflow in the event of power interruption. As the pump starts rotation, pressure builds and opens the aperture. The broad range of operation modes allows the device to operate as either an LVAD at high speed (∼3,300 rpm) or an RVAD at low speed (∼2,300 rpm) without modification and with the same electronic hardware. During operation, the axial movement of the rotor causes the aperture at the impeller discharge to open and close along with the cardiac cycle. This increases pump output during systole and attenuates output during diastole, thereby helping to preserve pulsatility. The proposed UVAD design renders the pump truly universal and readily available (no adaptions needed) for use as either an LVAD or RVAD.

A magnetic force acting on the rotor will close the aperture if the rotor stops spinning, preventing backflow during a power outage, controller failure, or while testing for weanability. The aperture feature also automatically slows its opening at low speed, allowing the UVAD to be used as an RVAD.

The UVAD rotor design consists of seven primary impeller blades, which influence hydraulic performance of the pump, and 12 secondary impeller blades, which assist in maintaining axial stability of the rotor during pump operation (19). The flow path between the operating regions of the primary and secondary impellers creates a journal bearing lubricated by blood. An aperture (located adjacent to and downstream of the primary impeller) acts as a pressure-regulating flow restrictor. This aperture, combining with a volute that is axially offset, are key characteristics that allow exceptional biventricular capability by the UVAD. This device also operates automatically and exhibits dynamic performance regulation, which is comparable to CFTAH (20). The UVAD’s rotor freely moves axially to respond to system pressure changes, which opens or closes the aperture feature located near the impeller discharge. The aperture operates like a differential pressure-regulating valve, adjusting to fluid pressure forces affecting the rotor, and conversely, magnetic forces generated by the motor (12, 13, 21, 22).

For this study, each UVAD was run at two different speeds for each condition; 3,800 rpm for the left pump, and 2,600 rpm for the right pump, was compared with 3,200 rpm for the left, and 2,000 rpm for the right.

### 2.3. Cannulation options for device connection

**FIGURE 3:**
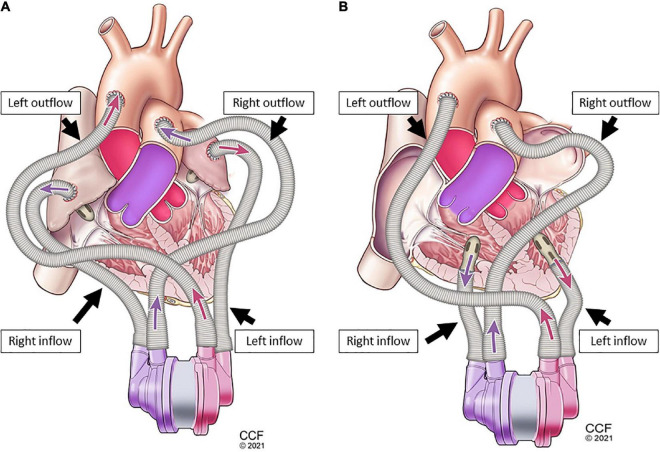
Single-device cannulation options continuous-flow total artificial heart (CFTAH): left and right ventricular inflow placement with corresponding aortic and pulmonary artery outflows **(A)**, and left and right atrial inflow placement **(B)** with corresponding aortic and pulmonary artery outflows.

**FIGURE 4:**
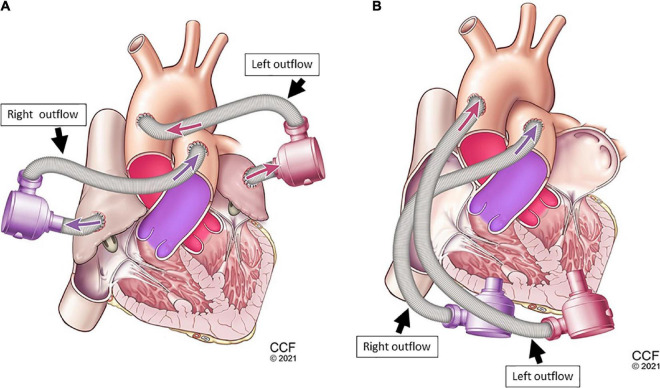
Dual-device cannulation options universal ventricular assist device (UVAD): left and right ventricular inflow placement with corresponding aortic and pulmonary artery outflows **(A)**, and left and right atrial inflow placement **(B)** with corresponding aortic and pulmonary artery outflows.

A biventricular test loop created HF scenarios utilizing dual pneumatic pumps (Abiomed AB5000™, Danvers, MA, USA) controlled by a double output driver. Inflows for one CFTAH (Figures 3A, B) and two UVADs (Figures 4A, B) were configured for both atrial and ventricular cannulations (15). Assessing BVADs occurred on a static test loop, which resulted in the generation of pressure-flow curves from several pump speeds. For each trial, the BVAD was operated over its entire range (i.e., device-specific motor speeds, aortic pressure ranging from 20 to 120 mm Hg), data was recorded, and pressure differentials were monitored and measured throughout the device.

In our feasibility study, the cannula had a longer length and smaller diameter, which resulted in an increase in flow resistance, requiring the pump to be operated at higher speeds than are typically used in a clinical setting. Ventricular cannulation was verified by measuring device inlet pressures with cannulae clamped proximally. *Via* fluid-filled pressure lines, hemodynamic data were recorded from both outflow cannulae. From fixed points on the outflow cannulae, near both pump outlets, total cardiac output was recorded.

## 3. Results

**FIGURE 5:**
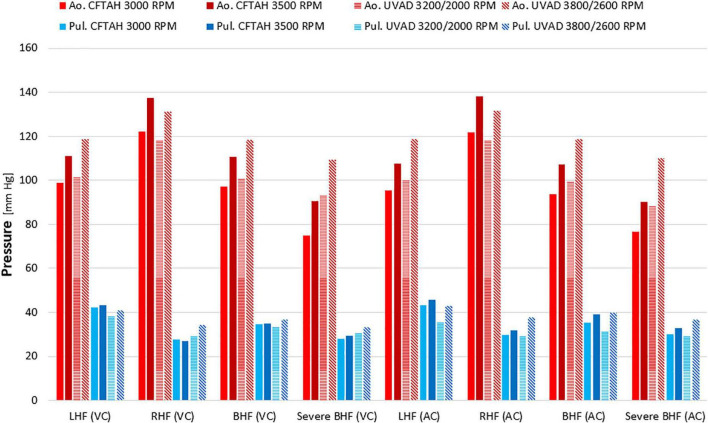
Arterial pressures at test conditions. Ao, aortic; Pul, pulmonary; LHF, left heart failure; RHF, right heart failure; BHF, biventricular heart failure; VC, ventricular cannulation; AC, atrial cannulation.

**FIGURE 6:**
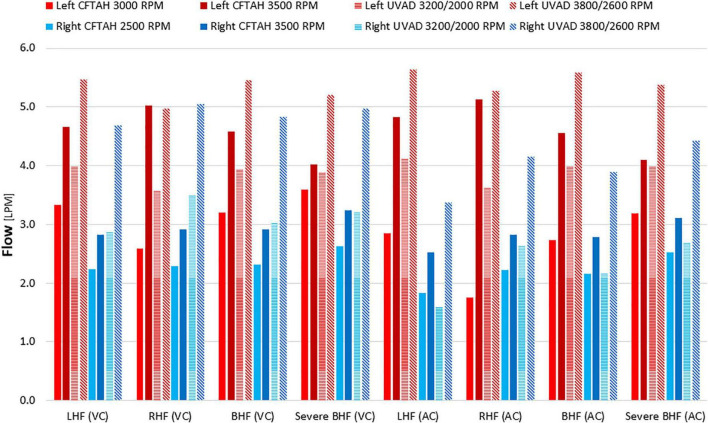
Right and left pump flow. LHF, left heart failure; RHF, right heart failure; BHF, biventricular heart failure; VC, ventricular cannulation; AC, atrial cannulation.

**FIGURE 7:**
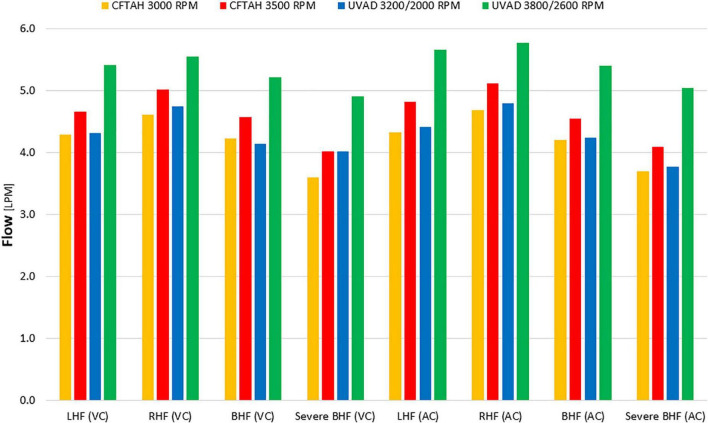
Total systemic flow. LHF, left heart failure, RHF, right heart failure, BHF, biventricular heart failure; VC, ventricular cannulation; AC, atrial cannulation.

From *in vitro* assessment in both device configurations, with an increase of BVAD speed, all flows (left pump, right pump, and total) increased as expected. Systemic arterial pressure (AoP) exhibited consistent dynamics between atrial and ventricular cannulation. Mean AoP dependably increased when pump speed increased and throughout the range of HF scenarios. Both CFTAH and dual UVAD configurations provided sufficient hemodynamic output within the tested speed range. AoP for all test conditions (Figure 5), covering a range of normal pressures, indicate that the pump capacities are sufficient for BVAD operation at the tested conditions. The corresponding pump flow rates are seen in Figure 6, and total systemic flow in Figure 7.

Corresponding atrial pressure differences show that an acceptable left and atrial pressure balance (LAP–RAP) between –5 and +10 mm Hg can be achieved with both pump configurations for moderate and severe BHF; however, the dual UVAD configuration resulted in better atrial pressure balance regulation over the range of test conditions and cannulation options (Supplementary Figure 1). Additionally, the atrial pressure balance for the CFTAH was not within the specified range for atrial cannulation in the LHF and BHF cases.

Heart valve flow (Supplementary Figure 2) was calculated by subtracting the pump flow from the measured total flow, and is an indication of the extent of the aortic and pulmonary valve opening. A calculated zero or negative valve flow indicates a valve remaining closed, or that the forward flow is less than the regurgitated flow, during the cardiac cycle. The pulmonary and aortic valves tended more toward closure at higher pump speeds and increased heart failure, as the pumps increasingly dominate the flow, bypassing the flow around the valves.

With the dual UVAD configuration, aortic pressure pulsatility (Supplementary Figure 3) tended to be somewhat greater than for the CFTAH. However, the pulmonary artery pulsatility was similar for both pump configurations, demonstrating that the UVAD and right CFTAH both have apertures at their impeller discharges.

In all cases with a single (CFTAH) and dual-device (dual UVAD) configurations, the pumps exhibited stable flow and motor current during all testing and performance evaluation. No power elevations, mechanical failures, or controller-related malfunctions occurred during these bench tests.

## 4. Discussion

This initial assessment of single CFTAH and dual UVAD device setups was conducted using unmodified device designs. Each of the two device configurations has its relative advantages when used for biventricular support. The CFTAH, as a single device, presents a less complicated system with fewer parts and the simple control of one speed. Results indicate that atrial pressure regulation is acceptable for cases of relatively matched BHF, but is challenged for cases of moderate left or moderate right failure.

There are reasons for challenged atrial regulation. Although the CFTAH regulates atrial pressure difference as a total heart, as a BVAD with ventricular cannulation, the atrial pressures are obscured from the pump inlets. Also, the CFTAH regulates better when the left and right pump flows are about equal, which may not be the case for the BVAD application, where the flow through the aortic and pulmonary valves are variable. The right pump aperture in a single device configuration (CFTAH) would control the self-regulation since the process is enabled through mechanical architecture of the device. With dual-device setup, the devices do not directly communicate with each other, since each device architecture is independently controlled and regulated for each device. The interaction of the dual-device configuration in presence of native heart valves makes the system regulation functional. The capacity of the CFTAH (23) which has been used at 9 L/min during *in vivo* experiments, may be greater than needed for most patients, and a smaller version would be a more practical option. The pediatric CF total artificial heart (P-CFTAH) is 30% smaller than the CFTAH and may be suitable for smaller patients (14, 24).

Unlike using an outlet graft restriction to turn an LVAD into an RVAD, the novel aperture (mechanical regulator) of the UVAD as its restriction self-adjusts instantly to a given hemodynamic environment. In addition, the volute section is axially offset from the impeller to even-out pressures around the impeller, thus providing an axisymmetric pressure and flow distribution becomes possible. This allows the unique pump to operate over a wide range of flow/speeds without creating adverse secondary flow patterns. In the event of incipient suction, the inlet cannula pressure will suddenly drop and the aperture will reduce its opening, thereby decreasing the output of the pump and forestalling onset of suction until the controller can sense the event and reduce speed.

The relative disadvantage of the dual UVAD configuration in the current setup is having to use two devices and control two speeds (25). However, the configuration has the advantage of being able to meet a wider range of heart failure conditions, while maintaining atrial pressure balance. By adjusting the relative speeds, it can potentially compensate for the development of aortic or pulmonary valve regurgitation. The capability of independent, individual assessment of the left or right pump in dual device setup may represent an additional value for critical weaning strategies and/or estimation of ventricular recovery during support. Another advantage of the UVAD is that speed and current signals can be used to indicate flow, making the pump a virtual flow meter, which would be useful for speed control. The functional anatomy and pathogenesis of RV and LV failure is closely interconnected and either of these conditions affect the prognosis for the other (5, 22, 26, 27). Thus, the communication between circulatory assist devices is imperative for a successful operation in biventricular support (28). This is deemed possible only if identical device architecture and control interface is employed (25). More work with dual-device UVAD configuration supported by a single controller unit is currently pending.

Our *in vitro* evaluations assessed the BVAD performance and self-regulation features that are based on the original Cleveland Clinic artificial heart and universal assist device designs (20, 21, 23, 29). These early series of bench experiments with the single and dual MCS for BVAD support have demonstrated the intended hemodynamic output and device features and feasibility of BVAD support using both the one device and dual-device configurations.

Adequate pump flow is required for recovery of end-organ failure and outcomes in achieving optimal BVAD support. The cannulation options using ventricular and atrial types have been evaluated. In both BVAD applications (CFTAH or UVAD), the type of cannulation seemed to affect the pump performance and self-regulation. The BVAD system can be affected by the overall compliance of the pulmonary and systemic vascular resistance. Direct device interaction after placement in the chest with native ventricles (ventricular work), native valves (valve competence and opening/closure) would contribute to hemodynamics during BVAD support vs. replacement therapies when the ventricles are removed. Device orientation can be additionally affected by the type of cannulation. Dual-device support may favor the cannulation of atrial or ventricular chambers, using shorter, directly positioned inflow cannula. The versatility of the cannulation process could further be enhanced by using a dedicated range of interchangeable cannulae, specifically adjusted for the type of support (left or right) and site of insertion (thinner atrial wall, or thicker ventricular tissue). Types of cannulation would potentially increase or reduce the pressure loss and position the BVAD closer to or farther from the native heart. This, in turn, would require device port orientation and flow evaluation of specific design changes made to the device inflow cannulae. Differences related to atrial and/or ventricular cannulae positioning and their clinical implications on thrombus formation, inadequate filling of the pump chambers, and optimal ventricular unloading must be assessed more extensively through specifically designed studies (30).

The options for clinical BVAD support are currently limited to two implantable LVADs or temporary device support (7, 9), and implantation modalities may vary, depending on the individual operator’s decision. Despite the efforts, there is currently no dedicated ventricular assist device approved for biventricular support; therefore, an LVAD implanted for BVAD support has to perform beyond the device design specifications (5). Rendering CF VADs universal could eliminate risks related to off-design performance of existing CF technology, and improve and standardize the clinical outcomes.

Operating a pump designed to be an LVAD as an RVAD would contribute to chronic “off design” flow patterns of flow recirculation caused by a relatively low rotor speed for a given flow rate (22). In UVAD design, the volute section is axially offset from the primary impeller to even out the pressures around the primary impeller, providing an axisymmetric pressure and flow distribution. This enables the UVAD to operate over a wide range of flows/rotor speeds without creating adverse secondary flow patterns that could lead to thrombus formation (31–33). This broad range of operating conditions allows the UVAD to function as either an LVAD at higher speeds/higher pressures, or as an RVAD with lower speeds/lower pressures, without modification (19).

The electronic device controllers used in the dual UVAD configuration for this experiment are independent and do not communicate with each other. Two controllers capable of working as a combined system with a more sophisticated control scheme could bring additional value for BVAD support and better self-regulation of hemodynamics. A combined controller unit could run both MCS devices in concert with synchronized outputs (25), and reduce the chances of controller failure (34). In a dual-device approach, with functional cardiac valves in place, the CF BVAD systems perform in more forgiving fashion than with the incompetent native valves. In cases of severe BHF, the aortic and/or pulmonary valves may remain closed some or all of the time. With two conventional CF pumps, more sophisticated control assessment may be needed for speed adjustment and atrial pressures balance. With blood pump-enabled self-regulation through incorporated pressure regulators, the problem may be solved due to instant mechanical response of the pump architecture to volume overload, pre-suction conditions and atrial balance.

In comparison with the single-device approach (CFTAH), the use of more than one device (dual UVAD) requires a dedicated device controller for each pump, which independently fine-tunes each blood pump according to the hemodynamic criteria. Rendering this dual-controller architecture into a single-controller unit can be addressed by designing a single-driver unit that could automatically synchronize outputs for both devices, running both MCS devices in concert (25), and further reducing the chances of controller failure (34).

This study is limited by the range of conditions that are possible to simulate on the circulatory loop, as well as in the investigation of control schemes and variability of potential clinical scenarios requiring BVAD. The pulsatility augmentation feature of the dual UVAD vs. CFTAH has not been performed. The automatic shut-off function of the UVAD has not been assessed, and will be tested in a specifically designed study. The validation of suction conditions and optimization of these parameters by a device controller were outside the scope of the current effort and will have to be further evaluated.

## 5. Conclusion

This study demonstrated the feasibility of supporting BHF using either of the two circulatory support configurations: the single-device (CFTAH with a single controller) and dual-device (UVAD with two controllers) BVAD. This initial characterization of single and dual-device circulatory support for BHF demonstrated device performance, flow, and pressure balancing over a range of simulated hemodynamic conditions. For moderate and severe BHF, both configurations performed well with regard to hemodynamic output and atrial pressure regulation. However, the UVAD configuration provided better atrial pressure balance under all tested HF conditions, as well as improved aortic pressure pulsatility.

Future directions should consist of technology characterization and optimizations in specific HF populations, with intent to elaborate on the most appropriate hemodynamic patient profile for a dedicated device configuration.

## Data availability statement

The original contributions presented in this study are included in this article/Supplementary material, further inquiries can be directed to the corresponding author.

## Author contributions

JK and DH: study design, data collection, analysis, and manuscript preparation. CM and CF: data collection, analysis, and manuscript preparation. AP: device development and data collection. BK: device development. TK: data analysis and manuscript preparation. KF and RS: study design and data analysis. All authors contributed to the article and approved the submitted version.

## Supplementary material

Supplementary Figure 1 Atrial pressure balance. LAP, left atrial pressure; RAP, right atrial pressure; LHF,left heart failure; RHF, right heart failure; BHF, biventricular heart failure; VC, ventricular cannulation; AC, atrial cannulation.

Supplementary Figure 2 Left (aortic) and right (pulmonary) heart valve flows. LAP, left atrial pressure; RAP, right atrial pressure; LHF, left heart failure; RHF, right heart failure; BHF, biventricular heart failure; VC, ventricular cannulation; AC, atrial cannulation.

Supplementary Figure 3 Arterial pressure pulsatility. Ao, aortic; Pul, pulmonary; LHF, left heart failure; RHF, right heart failure; BHF, biventricular heart failure; VC, ventricular cannulation; AC, atrial cannulation.
