# Supplementary material for: Biventricular circulatory support using single-device and dual-device configurations: Initial pump characterization in simulated heart failure model
Source: Front Cardiovasc Med. 2023 Feb 22;10:1045656. doi: 10.3389/fcvm.2023.1045656 (PMC9994815; doi:10.3389/fcvm.2023.1045656)
Supplement: Supplementary Figure 1 — Atrial pressure balance. LAP, left atrial pressure; RAP, right atrial pressure; LHF,left heart failure; RHF, right heart failure; BHF, biventricular heart failure; VC, ventricular cannulation; AC, atrial cannulation. [file Presentation_1.pptx]

## Slide 1
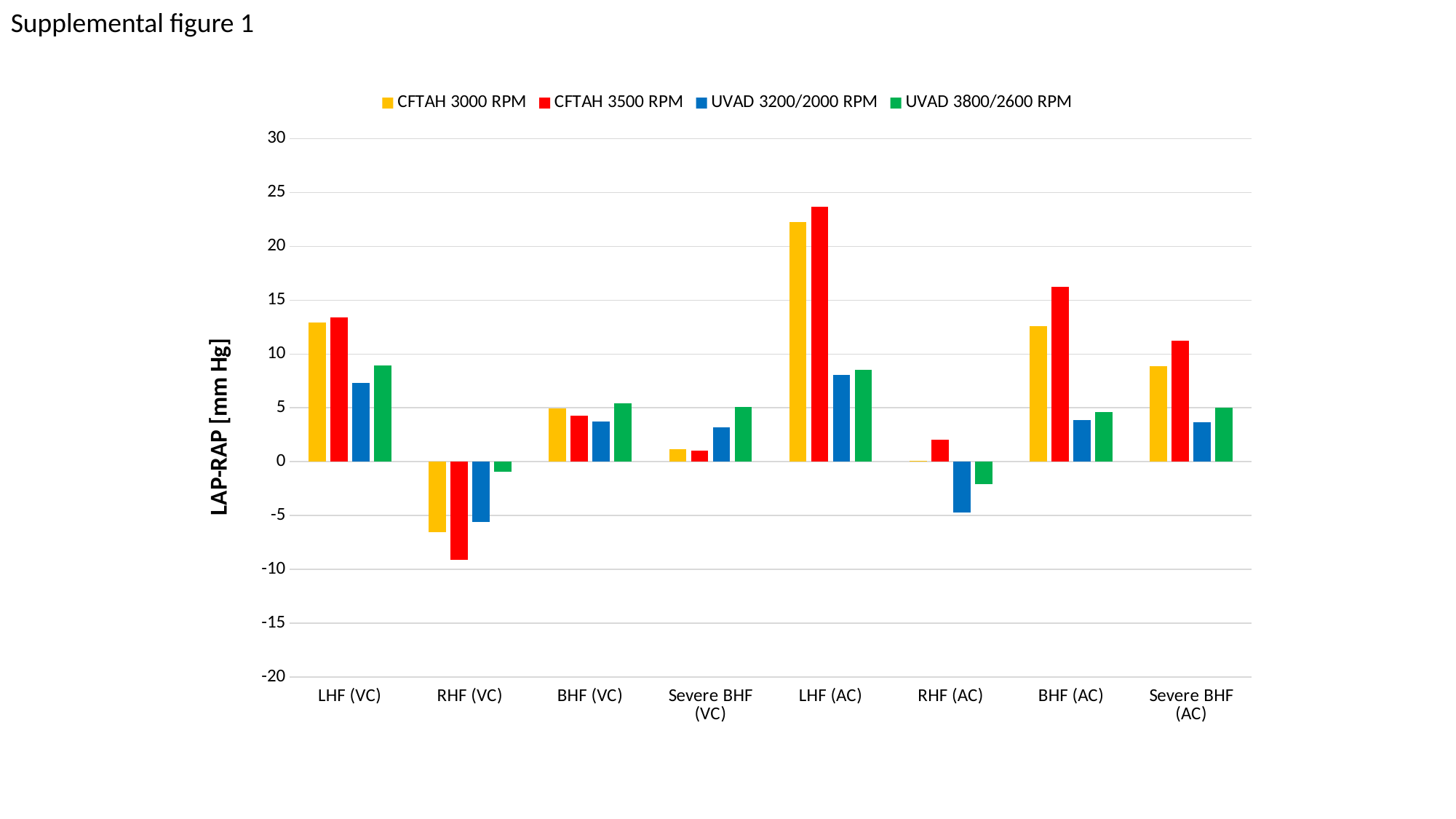

Supplemental figure 1
### Chart
| Category | | | | |
|---|---|---|---|---|
| LHF (VC) | 12.935599999999999 | 13.422099999999999 | 7.294122143000001 | 8.927624245 |
| RHF (VC) | -6.537300000000001 | -9.120899999999999 | -5.632502564 | -0.9512840916 |
| BHF (VC) | 4.9506 | 4.2417 | 3.7053729850000003 | 5.4524323557 |
| Severe BHF (VC) | 1.1450000000000014 | 1.0494000000000003 | 3.2103237459999994 | 5.092390984000001 |
| LHF (AC) | 22.2479 | 23.7147 | 8.023763401 | 8.556457523 |
| RHF (AC) | 0.06130000000000102 | 2.0471000000000004 | -4.729836206 | -2.0860935800000004 |
| BHF (AC) | 12.562500000000002 | 16.208599999999997 | 3.8454724099999993 | 4.618116612 |
| Severe BHF (AC) | 8.8487 | 11.204 | 3.630577413000001 | 5.018702560000001 |

## Slide 2
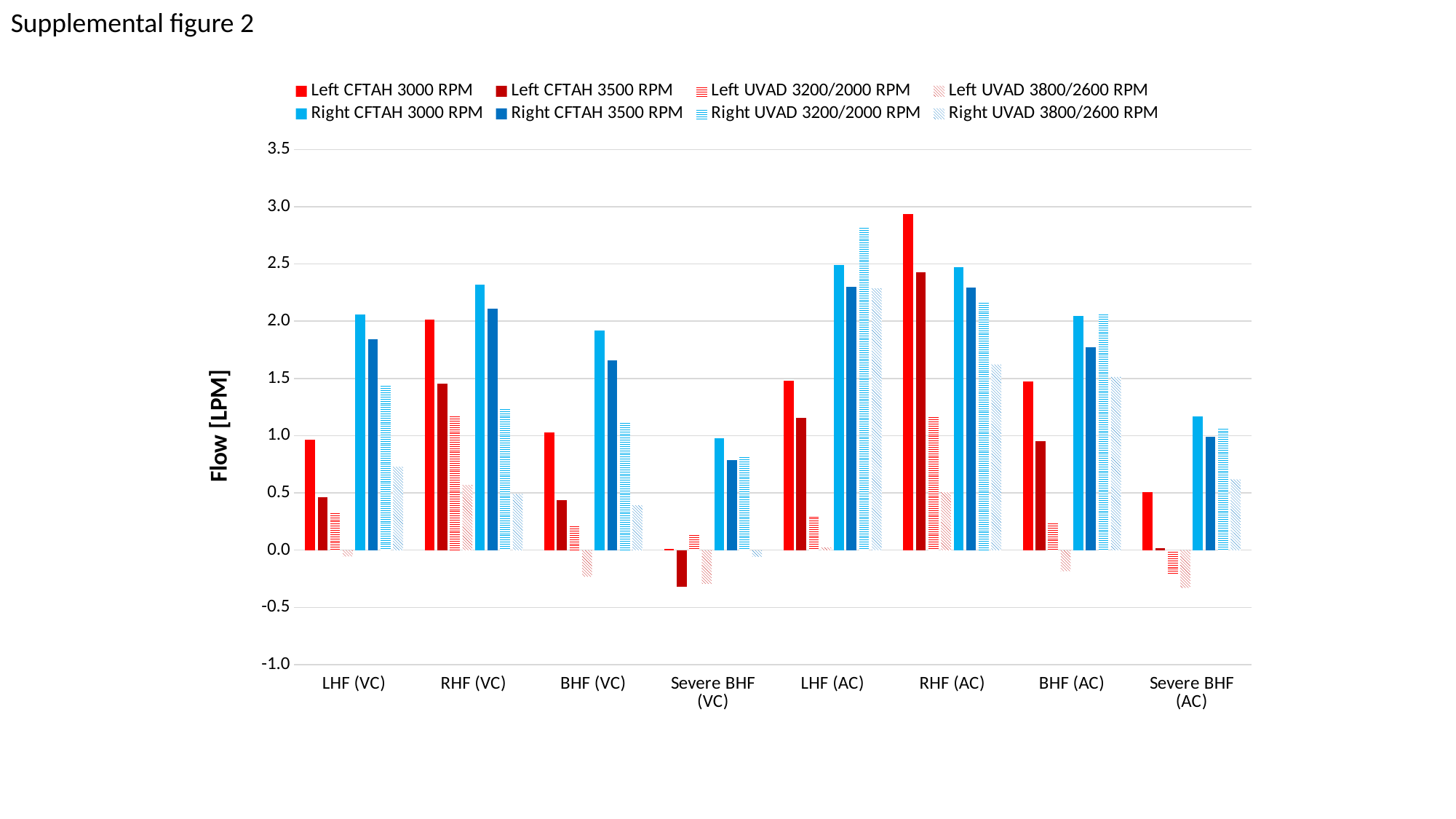

Supplemental figure 2
### Chart
| Category | | | | | | | | |
|---|---|---|---|---|---|---|---|---|
| LHF (VC) | 0.9667000000000003 | 0.46469999999999967 | 0.3393723129999997 | -0.05409543100000036 | 2.0606000000000004 | 1.8435000000000001 | 1.448998224 | 0.7278877399999999 |
| RHF (VC) | 2.0146 | 1.4541000000000004 | 1.18279404 | 0.5692313760000003 | 2.3217 | 2.1094000000000004 | 1.2486327150000003 | 0.4909940370000001 |
| BHF (VC) | 1.0298999999999996 | 0.4348000000000001 | 0.22189725299999985 | -0.231874876 | 1.9156999999999997 | 1.6589 | 1.1267660569999998 | 0.3910056939999995 |
| Severe BHF (VC) | 0.011499999999999844 | -0.3169000000000004 | 0.14483958799999996 | -0.2938551010000001 | 0.9803000000000002 | 0.7837000000000001 | 0.8250297099999999 | -0.057547452000000554 |
| LHF (AC) | 1.4773999999999998 | 1.1526999999999998 | 0.3097346099999996 | 0.02468755100000042 | 2.4928999999999997 | 2.3026999999999997 | 2.830549189 | 2.28657069 |
| RHF (AC) | 2.9364 | 2.4288 | 1.1751871939999998 | 0.5056403930000002 | 2.4713999999999996 | 2.2935999999999996 | 2.176471473 | 1.622195917 |
| BHF (AC) | 1.4729 | 0.9493000000000005 | 0.24831712000000028 | -0.18386452200000036 | 2.0472 | 1.7707000000000002 | 2.072184635 | 1.5130081080000002 |
| Severe BHF (AC) | 0.5042 | 0.01529999999999987 | -0.2154570819999999 | -0.32989193299999986 | 1.1694 | 0.9904999999999995 | 1.078250535 | 0.6180118380000001 |

## Slide 3
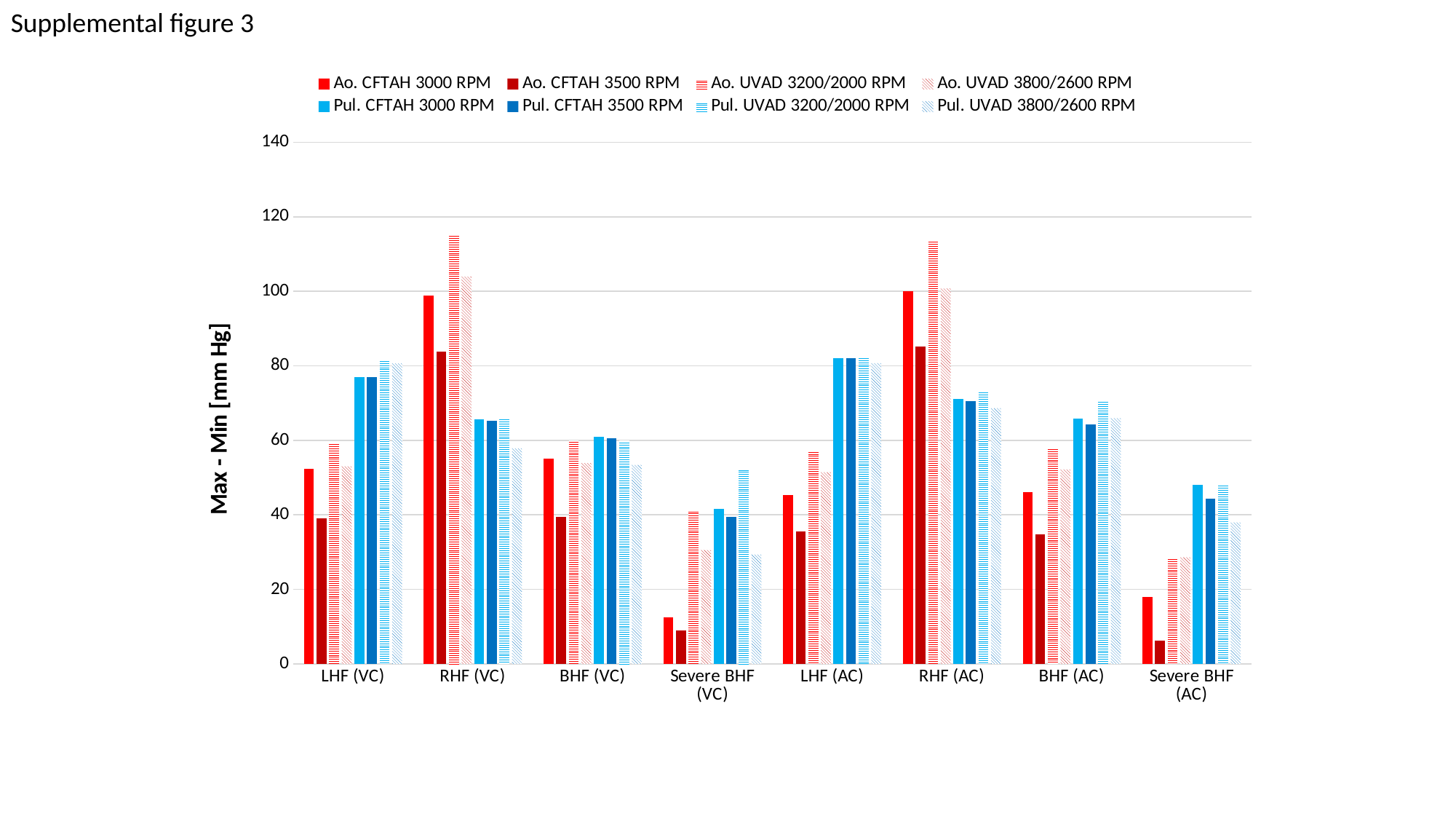

Supplemental figure 3
### Chart
| Category | | | | | | | | |
|---|---|---|---|---|---|---|---|---|
| LHF (VC) | 52.339299999999994 | 39.013400000000004 | 59.45171825999999 | 53.02923538 | 76.95389999999999 | 77.0709 | 81.79473648999999 | 80.63988149000001 |
| RHF (VC) | 98.8453 | 83.8549 | 115.29833173 | 103.95725339 | 65.5555 | 65.22999999999999 | 66.064674758 | 57.86271723 |
| BHF (VC) | 55.065 | 39.3904 | 60.064786780000006 | 53.868947320000004 | 60.93110000000001 | 60.5467 | 59.936029180000006 | 53.38013194 |
| Severe BHF (VC) | 12.548099999999991 | 9.066699999999997 | 41.424677020000004 | 30.564072730000007 | 41.668099999999995 | 39.4983 | 52.554391710000004 | 29.365019669999995 |
| LHF (AC) | 45.320800000000006 | 35.4862 | 57.419342060000005 | 51.43690604000001 | 82.00789999999999 | 81.96509999999999 | 82.588451113 | 80.71486378 |
| RHF (AC) | 99.95950000000002 | 85.09939999999999 | 113.81279211000002 | 100.74729248 | 71.1623 | 70.5214 | 73.341820834 | 68.62271539 |
| BHF (AC) | 46.0753 | 34.797200000000004 | 58.10719109 | 52.18100452 | 65.7579 | 64.2865 | 70.797984225 | 65.95683944000001 |
| Severe BHF (AC) | 17.915300000000002 | 6.282600000000002 | 28.6154466 | 28.59808910000001 | 47.977199999999996 | 44.4264 | 48.332027135 | 37.95004209 |
